# Laparoscopic vs. open inguinal hernia repair in Central Asia: a systematic review of technological readiness and safety frameworks

**DOI:** 10.3389/fsurg.2026.1761289

**Published:** 2026-05-13

**Authors:** Yerlan Akkaliyev, Maksut Kamaliev, Merkhat Akkaliyev, Elvira Kokayeva, Oxana Tsigengagel

**Affiliations:** 1 Department of Surgery, Medical Center Hospital of the Presidential Affairs Administration of the Republic of Kazakhstan, Almaty, Kazakhstan; 2 Department of Health Management, Kazakhstan Medical University “Kazakhstan School of Public Health”, Almaty, Kazakhstan; 3 Department of Surgical Disciplines, NCJSC Semey Medical University, Semey, Kazakhstan; 4 Department of Internal Medicine, Faculty of Medicine and Healthcare, Al-Farabi Kazakh National University, Almaty, Kazakhstan; 5 Department of Epidemiology and Biostatistics, Astana Medical University, Astana, Kazakhstan

**Keywords:** Central Asia, inguinal hernia, patient safety, surgical education, technological readiness, value-based healthcare

## Abstract

**Background:**

Inguinal hernia repair is the most frequently performed general surgical procedure worldwide. While the transition from open to laparoscopic techniques (TAPP/TEP) is a success story in high-income nations, developing healthcare systems in Central Asia face a complex reality where the adoption of technology often outpaces surgical training.

**Objectives:**

We aimed to systematically review the evidence on hernia repair outcomes, specifically interrogating the trade-off between short-term recurrence and long-term quality of life (chronic pain). Additionally, we sought to evaluate the critical role of surgeon “technological readiness” in settings with limited resources.

**Methods:**

We conducted a systematic review in strict adherence to PRISMA 2020 guidelines. Searching PubMed, Scopus, and the Cochrane Library (2004–2026), we identified studies comparing laparoscopic vs. open repair and assessing surgical education in Low- and Middle-Income Countries (LMICs). Eligibility was defined via the PICOS framework, focusing on adult patients, recurrence/pain outcomes, and safety profiles in RCTs and observational studies.

**Results:**

A total of 34 studies were synthesized. The evidence confirms that while laparoscopic repair significantly reduces the risk of chronic pain compared to the Lichtenstein technique, this benefit is strictly contingent upon high surgical volume (>60 procedures per surgeon). Crucially, long-term registry data indicate that 42.5% of recurrences appear more than 10 years post-surgery a timeframe often ignored in short-term safety assessments. In the LMIC context, open repair remains a robust, cost-effective standard where simulation-based training is absent.

**Conclusion:**

While laparoscopic repair can be safely implemented in LMICs with dedicated training, the direct transposition of international guidelines without local adaptation poses safety risks. We propose a “Safety-First” Value-Based Decision Framework, emphasizing that context-sensitive implementation and institutional readiness must take precedence over technical novelty to ensure patient value.

## 1 Introduction

Inguinal hernia repair is the most frequent operation in general surgery globally, representing a massive burden of disease. The shift from tissue repairs (like Bassini and Shouldice) to the Lichtenstein tension-free mesh technique was a game-changer, effectively dropping recurrence rates to below 4% (1, 2). However, the arrival of minimally invasive techniques, specifically Transabdominal Preperitoneal (TAPP) and Totally Extraperitoneal (TEP) repairs, has sparked an ongoing debate about what constitutes the “ideal” surgical standard (3, 4).

International bodies like the European Hernia Society (EHS) generally recommend laparo-endoscopic approaches for primary inguinal hernias in men. Yet, this recommendation assumes the surgeon has specific expertise, advanced resources, and a stable supply chain (5, 6). In wealthy nations, these prerequisites are handled through national registries and standardized simulation training. But the situation in Central Asia, and specifically Kazakhstan, is quite different. Here, the rush to adopt advanced laparoscopy is often driven by market competition and prestige, rather than by systemic readiness or strict safety protocols.

Currently, the spectrum of inguinal hernia repair in Central Asia reflects a marked rural–urban disparity (7). Based on regional institutional data and clinical observations across Kazakhstan, open Lichtenstein repair remains the undisputed workhorse, accounting for an estimated 75%–85% of all inguinal hernia procedures nationwide, predominantly in district and regional hospitals. Conversely, in major urban tertiary centers (e.g., Almaty and Astana), laparoscopic approaches are being increasingly adopted. Among these, the Transabdominal Preperitoneal (TAPP) technique appears to be more frequently utilized than the Totally Extraperitoneal (TEP) approach, largely due to its shorter learning curve, more familiar anatomical landmarks, and the lack of a requirement for expensive dissecting balloons (3, 4). However, the exact scale of this transition remains undocumented, highlighting the urgent need for a centralized national registry to accurately capture procedural trends.

From an epidemiological perspective, Central Asia is facing an aging population, which correlates with an increasing prevalence of surgical comorbidities. This shift is occurring alongside significant logistical constraints, as rural access to advanced surgical centers remains limited. The absence of robust local data further impedes the objectives set forth by the Lancet Commission on Global Surgery, which advocates for data-driven national surgical planning in low- and middle-income countries (LMICs) (8). As highlighted by Kingsnorth, hernia management strategies in developing regions cannot be mere replicas of high-income models, as the economic and educational landscapes are fundamentally distinct (9).

At present, clinical decision-making in the region is frequently skewed toward immediate outcomes, such as cosmetic appearance or reduced hospital stay, while often overlooking the long-term risk of late recurrence or the acute safety hazards of utilizing complex instrumentation without adequate proficiency. In this review, we critically examine the risks associated with the unadapted application of international guidelines to the Central Asian context. We contend that the absence of recurrence is no longer a sufficient metric for success. Instead, we propose a transition toward a Value-Based Decision Framework centered on “Technological Readiness” and institutional accountability (10–12).

## 2 Materials and methods

### 2.1 Search strategy

To ensure a comprehensive evaluation, two independent reviewers conducted a systematic search of major electronic databases (PubMed, Scopus, and the Cochrane Library) for literature published from January 2004 to February 2026. The search strategy employed key MeSH terms, including “Inguinal Hernia,” “Laparoscopy,” “Herniorrhaphy,” “Learning Curve,” “Patient Safety,” and “Surgical Education,” utilized in relevant Boolean combinations. Additionally, we manually screened the reference lists of included articles to identify further relevant studies, with a specific focus on global surgery and healthcare in Low- and Middle-Income Countries (LMICs). This review was conducted in adherence to PRISMA 2020 guidelines.

### 2.2 Eligibility criteria

Eligibility was determined based on the PICOS framework. We included studies if they: (1) provided comparative data on clinical outcomes (pain, recurrence, complications) between open and laparoscopic repair; (2) analyzed the learning curves and specific training requirements for TAPP/TEP; or (3) critically discussed surgical safety, cost-effectiveness, and infrastructure barriers in developing regions (13). The selection was strictly limited to English-language publications. Case reports, editorials, and animal studies were excluded to maintain the hierarchy of evidence.

### 2.3 Data extraction and synthesis

**Figure 1:**
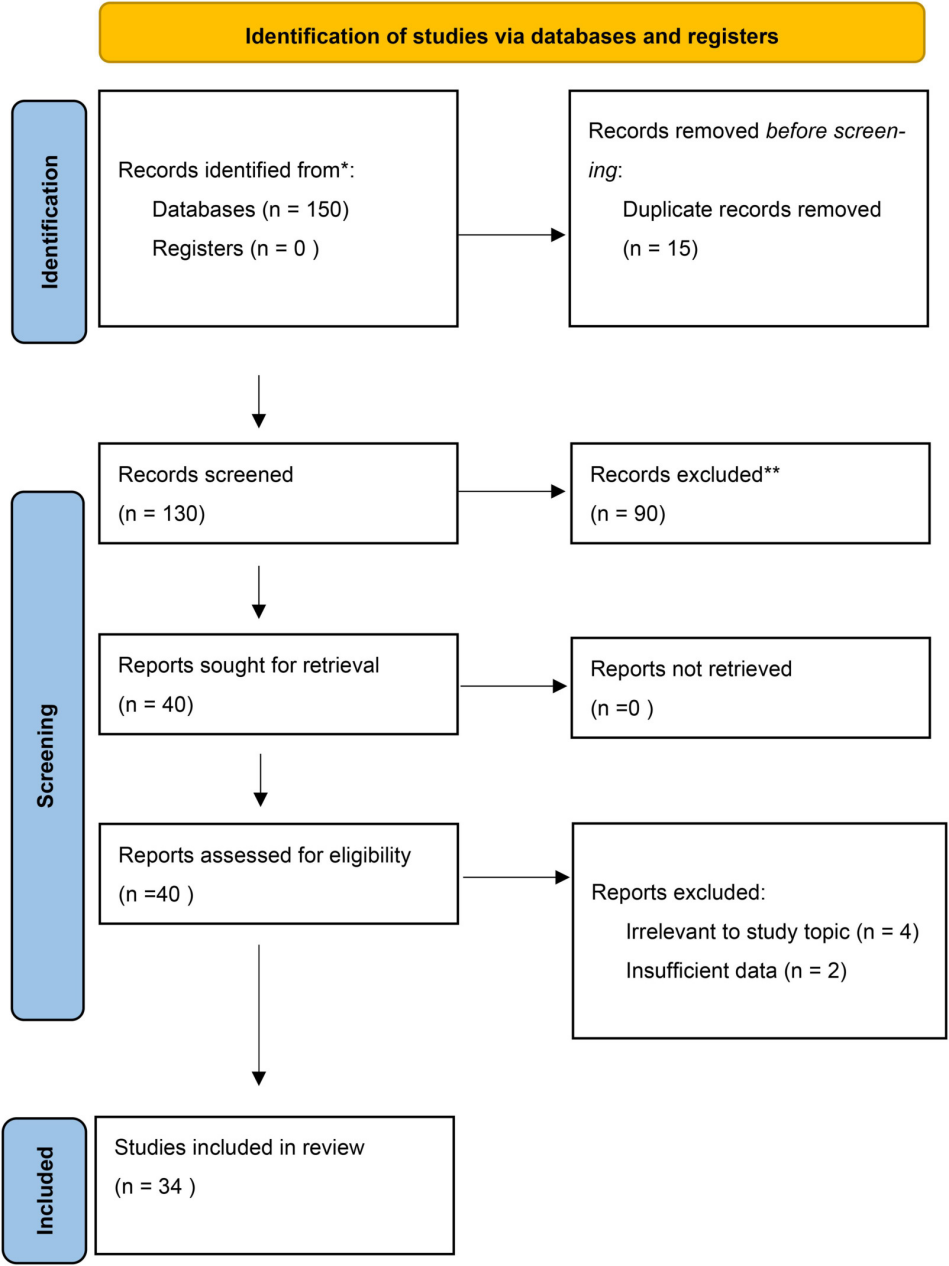
Flowchart of the study based on the PRISMA guidelines.

We systematically extracted data across key themes: recurrence rates, chronic pain incidence, learning curve thresholds, cost-effectiveness, and logistical barriers. Given the heterogeneity of the included studies, which ranged from Randomized Controlled Trials (RCTs) to registry analyses and observational studies, a quantitative meta-analysis was not feasible for all outcomes. Instead, we employed a narrative synthesis to integrate the findings into a cohesive safety framework. The detailed selection process is illustrated in the PRISMA flow diagram (Figure 1).

### 2.4 Quality assessment

Given the heterogeneity of the included studies, we adopted a qualitative risk of bias assessment. Systematic reviews were evaluated based on AMSTAR 2 principles, prioritizing those with rigorous search strategies (e.g., Cochrane reviews). Observational studies were critically appraised for selection bias and follow-up duration, with higher weight given to registry analyses with >5 years of follow-up.

## 3 Results

### 3.1 Study selection and characteristics

Our systematic search initially identified 145 records. Following a rigorous screening process to remove duplicates and irrelevant entries, we assessed 40 full-text articles. Eight were excluded for lacking specific relevance to the challenges of developing regions. Ultimately, 34 studies met our strict inclusion criteria and were included in the qualitative synthesis (Figure 1).

The evidence base is diverse, ranging from high-level Randomized Controlled Trials (RCTs) and systematic reviews to comparative observational studies that reflect real-world practice. Key characteristics of these studies are summarized in Table 1.

**Table 1:** Characteristics of key studies included in the systematic review.

Author/Year	Study design	Comparison/focus	Key findings/outcome
Outcomes & safety			
Andresen et al., 2024 (4)	Cochrane Systematic Review	TAPP vs. TEP (RCTs)	Updated evidence shows no significant difference in recurrence between TAPP and TEP, but TAPP carries a higher risk of visceral injury.
Sun et al., 2020 (13)	Meta-Analysis	Lap vs. Open	Laparoscopy reduces chronic pain and wound infection but significantly increases operative time.
Köckerling et al., 2015 (14)	Registry Analysis	Long-term follow-up	42.5% of recurrences occur >10 years post-surgery, highlighting the flaw of short-term safety studies.
Training & education			
Sivakumar et al., 2023 (15)	Meta-Analysis	Learning Curve	Operative time stabilizes at 35–40 cases, but safety plateau (recurrence/complications) requires >60 procedures.
Pelly et al., 2022 (16)	Systematic Review	Simulation Models	Simulation training significantly improves skills transfer to the OR compared to standard residency training.
Health systems & economics			
Stabilini et al., 2018 (17)	Consensus Statement	Institutional Standards	Defined criteria for “Hernia Centers” (high volume, mandatory registry) are essential to guarantee safety.
Shillcutt et al., 2010 (18)	Cost-Effectiveness	Ghana (LMIC context)	Open tension-free mesh repair is highly cost-effective in low-resource settings compared to laparoscopy.
Picciochi et al., 2025 (7)	Cohort Study	55 LMICs	First-referral hospitals in LMICs can safely perform hernia surgery but lack laparoscopic resources and mesh.

### 3.2 Synthesized findings: recurrence and the long-term perspective

**Figure 2:**
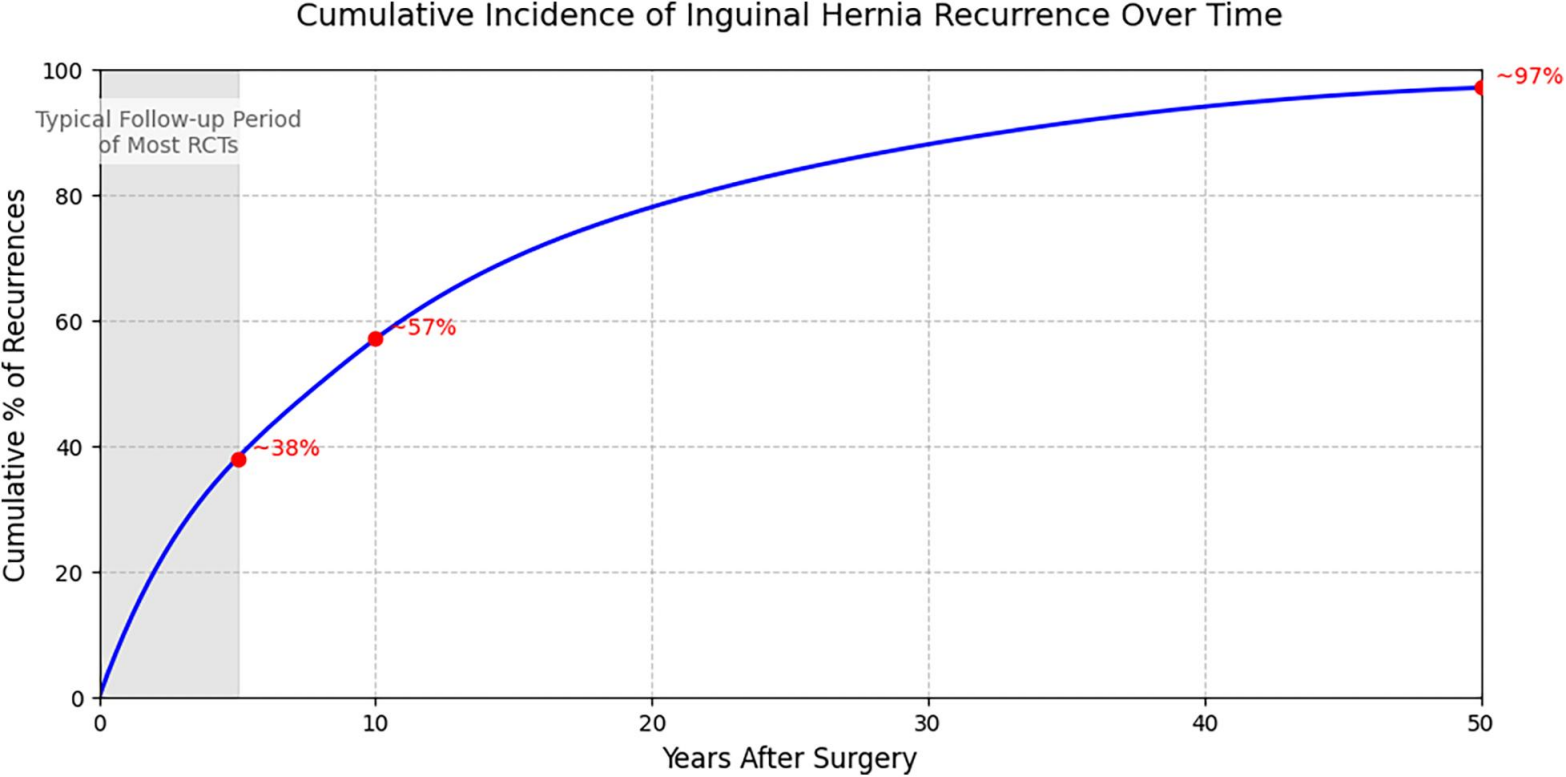
Cumulative incidence of inguinal hernia recurrence over time based on long-term registry data. The shaded grey area represents the typical 1- to 5-year follow-up period of most randomized controlled trials (RCTs), which captures only ∼38% of all potential lifetime recurrences. Adapted from Köckerling et al. (14).

For generations of surgeons, the absence of recurrence was the undisputed “gold standard” of success. However, our synthesis of long-term data suggests that relying solely on this metric is fundamentally flawed when evaluating modern technology. A landmark analysis of the Herniamed registry highlights a critical finding: approximately 42.5% of recurrences occur more than 10 years after surgery (14). This reality undermines the safety assurances of numerous clinical trials that rely on short 1- to 5-year follow-up periods (Figure 2), as they fail to capture the lifetime risk of mesh shrinkage or fixation failure.

While early meta-analyses confirmed the general superiority of mesh-based repairs (1), modern evidence indicates that the long-term success of the specific approach is highly dependent on the setting. Expert centers consistently demonstrate superior long-term outcomes for laparoscopy; however, real-world data in low-volume settings suggests that the technical reproducibility of open repair may offer more consistent results where surgical expertise is variable (20). For a developing healthcare system, relying on short-term data to validate complex laparoscopic programs creates a latent risk of late failures caused by inadequate technique during the learning phase.

### 3.3 Synthesized findings: chronic pain as the new priority

Today, the clinical conversation has shifted. Modern literature suggests that Chronic Postoperative Inguinal Pain (CPIP) is often more debilitating to a patient's quality of life than a recurrence. Systematic reviews consistently indicate that laparoscopic repair significantly reduces the risk of CPIP compared to the Lichtenstein technique (3, 4, 21). Specifically, Sun et al. (2020) confirmed lower rates of chronic pain and wound infection (14), while Eklund et al. (2010) reported that five years' post-surgery, moderate-to-severe pain was far less frequent after TEP (1.9%) than open repair (19%) (22).

However, this “pain advantage” is not automatic; it is strictly technique-dependent. Theoretically, laparoscopy works better because the mesh sits in the preperitoneal space, avoiding the groin nerves. But as Peitsch (2014) noted, these excellent results are typically achieved only in high-volume centers (23). In inexperienced hands, the risk of nerve entrapment remains a concern, specifically when traumatic fixation (tackers) is utilized. While modern guidelines advocate for self-gripping meshes or glue, tackers remain common in resource-constrained settings due to supply chain and cost limitations (6).

We must also consider the safety profile highlighted by the most recent Cochrane update (Andresen et al., 2024). While TAPP and TEP techniques share similar recurrence rates, the TAPP approach carries an inherent theoretical risk of visceral injuries compared to extra peritoneal techniques, although recent Cochrane data suggest these events are rare (3). However, in a district hospital with limited backup, even a rare bowel or bladder injury represents a catastrophic event, reinforcing why advanced training is non-negotiable.

### 3.4 Synthesized findings: the steep learning curve

Laparoscopy is not just a new technique; it is a distinct skill set with a formidable learning curve. We introduce the concept of “Surgeon Technological Readiness” as a non-negotiable safety standard.

While the learning curve for open Lichtenstein repair is relatively short (20–25 procedures), laparoscopy demands significantly more dedication. CUSUM analysis suggests a surgeon requires between 50 and 100 supervised procedures to reach a plateau where operative time and complication rates stabilize (3, 15, 24). A recent meta-analysis by Sivakumar et al. (2023) synthesized data from 43 studies and found that while operative time may stabilize after 35–40 cases, minimizing complications and recurrence requires a significantly longer learning phase of over 60 procedures (15).

Crucially, to ensure safety, resident autonomy in these complex procedures must be “well-earned” through objective performance metrics (25).

Being fast is not the same as being safe. Data indicates that while operative time may improve after 50 cases, the recurrence rate for laparoscopic repair continues to decrease until a surgeon has performed approximately 250 procedures (14). In Central Asia, where a district surgeon might perform only a limited number of hernia repairs annually, achieving true laparoscopic mastery could take over a decade. This creates a “dangerous valley” where surgeons operate perpetually in the high-risk phase of the learning curve (15), exposing patients to risks like bowel perforation that are virtually non-existent in open surgery.

### 3.5 Synthesized findings: the anesthesia factor

In the race for technological advancement, the humble choice of anesthesia is often overlooked, yet it is a critical safety factor. Laparoscopic repair necessitates general anesthesia, which poses specific risks to the elderly, including hemodynamic instability and cognitive dysfunction.

Comparative studies indicate that neuraxial (spinal) anesthesia, suitable for open repair but not laparoscopy, offers superior pain control, faster recovery, and less nausea in patients over 65 (26). This aligns with WHO-WFSA standards, which recommend matching the anesthetic plan to patient complexity (27). In developing regions, the capacity to perform open repair under local or regional anesthesia acts as a vital “safety valve” (19). As noted by Ndong et al. (2023), local anesthesia remains underutilized in developing countries despite its proven safety profile and low cost (7, 27).

### 3.6 Synthesized findings: economic implications

Adopting protocols from high-income nations without looking at the price tag is a dangerous strategy. In Low- and Middle-Income Countries (LMICs), imported consumables (mesh, tackers, disposables) for laparoscopy are expensive relative to local labor costs. Shillcutt et al. (2010) found that open mesh repair in Ghana was highly cost-effective, while the economic case for laparoscopy was much weaker (19). In extreme cases, some settings have even explored sterilized mosquito net mesh as a low-cost alternative to commercial products (28).

Furthermore, supply chain stability is a safety issue. A laparoscopic program requires a steady supply of CO2, sterile optics, and energy devices. If a district hospital faces a shortage, a surgeon might be forced to convert to open surgery or, worse, reuse disposable instruments, compromising sterility. O'Brien et al. (2021) emphasize that cost-effectiveness must be calculated locally; what is efficient in Europe may be fiscally unsustainable in Central Asia (29).

The barriers to widespread laparoscopic adoption in Central Asia extend beyond initial capital investment (30). The region faces significant workforce training deficits, compounded by limited access to structured mentorship programs and simulation-based training facilities (31). In addition, equipment availability is constrained by maintenance challenges; repairing a damaged laparoscope or energy device in rural district hospitals may take extended periods, temporarily disrupting surgical services (7, 8).

Cost constraints associated with single-use consumables (e.g., tackers and disposable trocars) contribute to the continued predominance of open techniques in many rural hospitals (19), thereby reinforcing the existing rural–urban disparity in access to minimally invasive surgery.

However, these economic barriers are not absolute. Evidence from various low- and middle-income country (LMIC) settings suggests that appropriately sterilized reusable laparoscopic ports can be safely utilized under standard infection control protocols, helping to reduce procedural costs without compromising sterility. In addition, the increasing adoption of advanced laparoscopic skills, such as intracorporeal suturing, as well as selective non-fixation strategies for mesh placement, may reduce reliance on expensive disposable fixation devices and improve the overall cost-effectiveness of laparoscopic repair in resource-limited environments (32).

## 4 Discussion

### 4.1 Synthesis of findings

The findings of this review highlight a critical disconnect between global high-tech trends and local safety requirements. While laparoscopic repair offers superior patient-reported outcomes (less chronic pain), these benefits are nullified if the procedure is performed by a surgeon within the “learning curve” danger zone (16). Based on the synthesized evidence, we propose a comparative framework to guide clinical practice in the region (Table 2).

**Table 2:** A modern comparative framework of inguinal hernia repair techniques.

Feature category	Open repair (lichtenstein)	Laparoscopic TAPP/TEP
Patient-centered outcomes		
Short-term recurrence (1–5 years)	Comparable (∼1.0%–4.0%) (1, 13)	Comparable (∼1.0%–4.0%) (4, 13)
Chronic postoperative pain (CPIP)	Higher incidence (∼10%–19%) (13, 21)	Significantly lower incidence (∼1.9%–5%) (13, 21)
Return to work/daily activities	Slower (∼14–21 days) (4)	Faster (∼7–14 days) (4)
Technological & system factors		
Technical complexity & cost	Low (Basic instrumentation, minimal consumables cost)	High (Tower; properly sterilized reusable or disposable ports) (31)
Surgeon learning curve	Short/Standard (∼25 cases)	Steep and Prolonged (>60 procedures required for safety) (15)
Patient safety risk	Minimal inherent risk to visceral organs	Low in experienced hands; theoretical risk of visceral/bladder injury during the learning curve (incidence ∼0.1% - 0.2%) (4)

Crucially, our synthesis reveals a significant divergence from the prevailing academic consensus in high-income nations. While guidelines from the HerniaSurge Group (6) prioritize the metabolic benefits of laparoscopy (less pain, faster recovery), our review suggests that in the specific context of Central Asia, these biological advantages are outweighed by systemic risks. This aligns with the broader Global Surgery 2030 framework proposed by Meara et al, which argues that surgical workforce density and safety must precede technological complexity (8). Therefore, unlike studies from Western Europe that focus on “refining” laparoscopic technique, our findings indicate that for Central Asia, the primary academic imperative is not technological adoption, but rather the establishment of safety moats, specifically, simulation credentialing and mandatory registries before widespread implementation.

### 4.2 Proposed framework

We advocate for a paradigm shift where the management of inguinal hernias is guided by Institutional Accountability rather than individual surgeon preference. To facilitate this transition, we propose the “Safety-First” Value-Based Decision Framework (Table 3). Our proposal is not a radical departure from clinical norms, but rather a call to adopt a proven, international standard of care. This approach directly mirrors the rigorous consensus established by the Italian Society of Hernia Surgery, which defined strict criteria for “Certified Hernia Centers” based on high surgical volume and mandatory registry participation (18). If such robust accountability is deemed a prerequisite for patient safety in Europe, we should aspire to an equivalent standard for our evolving healthcare system. The urgency of this institutional shift is further underscored by recent qualitative evidence from Kazakhstan, which reveals that health professionals across high-stakes specialties identify workforce training deficits and fragmented management as the primary systemic barriers to quality care (33).

**Table 3:** A proposed “safety-first” value-based decision framework.

Level of consideration	Key questions and guiding principles
1. Patient-centered value	*Key Questions*: What are the patient’s priorities? (e.g., fastest return to work vs. avoiding general anesthesia vs. lowest chronic pain risk). Clinical Context: Is the hernia primary or recurrent? Unilateral or bilateral?
	*Guiding Principle:* Align technique benefits with individual goals. Prioritize clinical safety, as patient satisfaction in the region is heavily driven by care quality (34). *Example:* Avoid laparoscopy in elderly patients with comorbidities who are suitable for local anesthesia.
2. Surgeon & team technological readiness	*Key Questions:* Does the surgeon have documented proficiency (>60 procedures) (16) beyond the initial learning curve? Is the entire operative team (nurses, technicians) familiar with the specific equipment required? *Guiding Principle:* The choice of a complex technique must be justified by proven competence. When in doubt, the safer, simpler option (Open Repair) is usually superior.
3. Institutional Accountability	*Key Questions*: Does the institution provide a structured, simulation-based curriculum? Are there clear credentialing policies? Is there a maintenance protocol (e.g., ability to repair energy devices)? Is there a registry system? *Guiding Principle:* The institution must provide a sustainable safety ecosystem. Ignoring training deficits or maintenance protocols is a failure of governance (33).

### 4.3 Limitations

This study is subject to several limitations. First, the majority of the high-level long-term data originates from Western registries, such as Herniamed (14), which may not perfectly reflect the specific patient demographics, comorbidities, or clinical environments of Central Asia. Second, the current lack of localized health-economic data in Kazakhstan restricts our ability to perform a precise cost-utility analysis. Finally, the narrative nature of this synthesis reflects the absence of regional randomized controlled trials, which currently precludes a statistical meta-analysis of local outcomes.

### 4.4 Future directions

To bridge the gap between theory and practice, the academic community in Central Asia must address specific knowledge gaps through a three-pronged approach:

*Educational Adaptation:* To break through “cultural resistance,” we must shift toward competency-based progression. Lorenz et al. (2021) demonstrated that structured training protocols are highly effective even in developing regions (25). Furthermore, emerging Augmented Reality (AR) tools utilizing standard hardware offer a viable, low-cost solution for training in resource-poor environments (8, 17).

*Economic Verification:* While Western data suggests that a quicker return to work renders laparoscopy cost-effective (3), this hypothesis requires local validation. A rigorous clinical-economic analysis based on national tariffs is essential to determine if this holds in an economy where imported consumables are costly relative to labor.

*Epidemiological Surveillance:* We advocate for the establishment of a national hernia registry, modeled after Herniamed (14). This is the only reliable method to track real-world long-term recurrence and chronic pain rates, moving from anecdotal evidence to data-driven quality control (7, 28).

Finally, we must move beyond the outdated “see one, do one” apprenticeship model. As confirmed by Pelly et al. (2022) in their systematic review, simulation is not merely an educational accessory; it significantly improves the transfer of skills to the operating room compared to traditional residency training (17). Therefore, surgical privileging should rely on objective simulation benchmarks rather than simple tenure. Specific research gaps identified are summarized in Table 4.

**Table 4:** Identified research gaps in herniology for Kazakhstan and Central Asia.

Research domain	Established global evidence	Identified gap in Kazakhstan & Central Asia
Health economics	Laparoscopy generally offers lower societal costs due to faster functional recovery, although direct procedural costs remain higher (3, 19).	Absence of localized evidence: A formal clinical-economic analysis based on national healthcare tariffs and regional societal productivity costs is currently missing.
Patient-centered outcomes	Minimally invasive techniques are linked to superior long-term Quality of Life (QoL) and a reduced incidence of chronic postoperative pain (13, 21).	Lack of context-specific data: Systematic studies utilizing validated QoL instruments (e.g., EuraHS or Carolinas Equation) on the Central Asian population have not been conducted.
Surgical education & safety	Structured, simulation-based training is the proven international standard for ensuring patient safety during the surgical learning curve (16, 30).	Systemic assessment gap: The current state of residency training, surgeon readiness for advanced techniques, and institutional credentialing standards remains unassessed in the region.
Clinical guidelines	International bodies (EHS and HerniaSurge) provide robust, evidence-based management protocols (5, 6).	Lack of local adaptation: Global guidelines have not been formally validated or adapted to address the logistical and infrastructure constraints of the local healthcare context.

## 5 Conclusion

The evolution of herniology in Kazakhstan and Central Asia stands at a critical juncture. Extensive evidence from various LMIC settings demonstrates that laparoscopic inguinal hernia repair can indeed be safely and effectively implemented when paired with dedicated training and high-volume practice. The technique itself is highly efficacious; however, the direct, unadapted transposition of Western protocols into resource-constrained environments requires refinement.

A balanced interpretation of the current evidence suggests that successful adaptation of surgical techniques depends primarily on the availability of equipment, institutional infrastructure, and surgeon expertise. Although contextual modification is essential, it does not imply any inherent unsuitability of minimally invasive approaches. Rather, the key challenge lies in context-sensitive implementation and the development of robust training systems to ensure patient safety.

Therefore, healthcare systems should move beyond the narrow focus on short-term recurrence and adopt a more comprehensive framework, incorporating the concept of Surgeon Technological Readiness. Such an approach would enable surgical innovation to deliver meaningful and sustainable value to patients.

## Data Availability

The original contributions presented in the study are included in the article/Supplementary Material, further inquiries can be directed to the corresponding author.
